# Cardiovascular Prevention: Migrating From a Binary to a Ternary Classification

**DOI:** 10.3389/fcvm.2020.00092

**Published:** 2020-05-22

**Authors:** Amy Lynn Doneen, Bradley Field Bale, David John Vigerust, Pierre P. Leimgruber

**Affiliations:** ^1^College of Medicine, Washington State University, Spokane, WA, United States; ^2^School of Medicine, Vanderbilt University, Nashville, TN, United States; ^3^School of Medicine, University of Washington, Seattle, WA, United States

**Keywords:** cardiovascular disease (CVD), plaque, atherosclerosis, inflammation, prevention

## Abstract

Migrating from a binary approach to risk assessment to a ternary model of disease identification allows for individualized, optimal disease management. Redefining the disease/inflammatory approach has been proven to identify, stabilize, and regress atherosclerosis while adding understanding to the progression of vascular disease. Our previously published results show the beneficial effect of comprehensive, evidence-based management on subclinical atherosclerosis and vulnerable plaque. We argue that this approach does not mitigate the value of utilizing standard risk factor identification, but rather augments it for the benefit of the individual patient.

## Introduction

Despite a growing wave of attention on the devastating consequences of sudden cardiac death and ischemic strokes, we still struggle with staggering and stagnant statistics within the United States (1). The latest data published March 2019 from the American Heart Association reveal that the incidence of cardiovascular disease (coronary heart disease, heart failure, stroke and hypertension) in adults ≥20 years of age is 48% overall, comprising 121.5 million people according to NHANES data from 2016. When excluding hypertension, the statistics remain alarming with 24.3 million people affected (2). The 2014–2015 projected direct and indirect cost of cardiovascular disease (CVD) is more than $351 billion ($213.8 billion in direct costs and $137.4 billion in lost productivity/mortality), overtaking the cost of any other diagnostic group. The projected annual CVD expense in 2035 is estimated to be 1.1 trillion dollars (2). “True healthcare reform will be realized only when we focus attention on disease prevention and not disease management,” former American Heart Association (AHA) president- Dr. Gordon Tomaselli (3). Currently 48% of US Adults have CVD, equating to 121.5 million. By 2025 it is projected that 130 million people in the US will have CVD. Appreciating the escalating economic burden and continued morbidity and mortality related to CVD, it is imperative that new innovations be considered to improve CV prevention. CV events place significant health and economic burden on our country. Improvements in our current system must be improved (1). We propose the first step is a re-classification of risk. A system that is directed at the individual patient and focuses on the optimization of precision healthcare. Healthcare must facilitate the uptake and deployment of prevention to improve health and lower costs (4).

Although the guidelines for treatment are based on a risk factor paradigm, plaque in the artery wall is the actual initial culprit of type 1 myocardial infarctions and systemic vascular events (5–7). Despite the latest modification of the Framingham Risk Factor Score analysis, the focus remains on the risk factors rather than the presence or absence of atherosclerosis (8). Even with the latest revision of the traditional risk factor analysis (9) to enhance treatment decisions, it fails to direct the initial evaluation for atheroma or vascular inflammation (10). Herein lies the conundrum that seems to elude repetitive modifications of the current risk factor paradigm assessment to reduce recidivism and stop the unexpected death and disability associated with vascular disease. The presence of an atheroma is essential in determining risk of having an atherothrombotic event therefore the evaluation of the presence of atherosclerosis is an indispensable element for risk assessment. As stated by Arbab-Zedeh et al., “atherosclerosis is essentially a condition sine qua non.” Therefore, assessing for atherosclerosis is fundamental for appropriate risk estimates (11).

## Binary to Ternary Classification

The guidelines for evaluating who might be “at risk” for a vascular event fail to look directly for the plaque, particularly the vulnerable plaque. The standard of care places individuals into those who have had a CV event (secondary prevention) and those who have not (primary prevention). Knowing if a patient is harboring silent, potentially deadly thickening in the artery wall, provides a new-found ability to triage treatment in a different way from the traditional risk factor paradigm (12). As eloquently articulated in the 2016 Ross Memorial Lecture in vascular biology, atherosclerosis is “triggered by the sub endothelial retention of ApoB-containing lipoproteins in focal areas of the arterial tree” (13). Tabas then continues to suggest “the ensuing events of complex cell biological processes (are) termed atherosclerosis.” Appreciating the atherogenic triad allows for the opportunity to understand how various causes of vascular risk can determined. The formation of atherosclerosis requires three essential factors: serum lipoprotein concentration, endothelial permeability and lipoprotein binding in the intima (14). As previously reported, there are many causes of atherosclerosis via interaction with this triad that are not part of a standard of care risk factor analysis, such as high risk periodontal pathogens (15).

The natural progression of vascular disease can begin in early adolescence as fatty streaks within the arterial wall (16). At the same time, it is important to realize that atherosclerosis and CVD are not unavoidable consequences of aging and there are occasions to intervene early in the disease progression and halt the atherosclerotic disease process (17, 18). Gradual, often silent expansion of extant lesions may eventually limit arterial blood flow. However, such slow evolving stenotic lesions are not usually the cause of acute coronary events; rather, either rupture or erosion of the endothelium overlying an atheroma leads to a thrombus. Often times, the thrombus causes an acute obstruction that results in a symptomatic ischemic event: heart attack or ischemic stroke. If a small thrombus is present, it may migrate distally, causing silent ischemia. Additionally, the thrombus may simply resolve, leading to a size progression in the underlying atheroma (19). This schema can happen in any artery and ultimately present as coronary, renal, intestinal, peripheral, or cerebral disease. With this understanding of the atherosclerotic disease process, identifying vulnerable plaques that are at high risk for causing a coronary event is a critical element for CV risk assessment, particularly in asymptomatic individuals who may be at risk for an event and yet be considered to be primary prevention in the traditional setting.

Based on the most recent work by Ahmadi et al., the approach discussed herein of identifying non-obstructive atherosclerosis to identify patients who are “at risk” for a cardiovascular event is well-supported and aims to redefine the traditional paradigm of primary and secondary assessment (20). However, we further support focusing treatment effects on the anti-inflammatory effect of identifying all root causes of vascular inflammation, often going beyond the hypothesis of simply obtaining LDL reduction from maximum lipid-lowering therapy. Incorporating inflammatory testing to evaluate the atherosclerotic activity provides the opportunity to individualize therapy to treat the *n* of 1 = the unique individual patient.

Applying a third category to CV risk assessment provides for a more accurate and individualized approach to treatment of the *n* of 1. Coupling structure (disease assessment) with inflammation provides a clear approach to treatment decisions.

Treatment for CVD is anchored in a classification system of primary or secondary. Currently, we are in a risk platform of a world of “have's” and “have nots,” simply meaning treatment decisions are based on whether or not the individual patient has had a heart attack, stroke, or CV intervention in the past. Secondary prevention labels a patient to have proven CVD either by having had an intervention for CVD or having had a CV event. These individuals are considered high risk and, while there are clear treatment guidelines for this category, the focus remains optimal LDL-C management and is often in some state of flux. The current risk category of “primary prevention” includes all individuals who have not proven they have CVD with an intervention or event. Therapy for this primary risk classification is determined by risk factors alone which is based on population data. It is well-known that for the individual person being considered the resulting “population” calculation of risk fails to identify a substantial number of people as high risk who go on to suffer a CVD episode of intervention or event. Likewise, it is also true that some people are incorrectly classified as high risk when using risk factors alone. The current risk-factor assessment is innately flawed as it fails to treat people individually based on the presence of arterial inflammation and non-obstructive disease. Often patients receive treatments that are unnecessary and many cause harm. Thus, our current classification of prevention is arguably potentially causing harm for many individuals in the “primary” category; some over treated and some under-treated.

A ternary classification system in light of our current knowledge about CVD and our current imaging technologies and inflammatory testing could enhance appropriate therapy decisions for the individual patient. It is known that having an atheroma is a condition sine qua non for having risk of a CV event (21). Therefore, assessing for an atheroma should be considered fundamental in any risk assessment. Historically, the risk factor classification was developed out of necessity because clinicians did not have easily assessible reliable, safe and cost-effective imaging technology to evaluate an individual patient for the presence of atherosclerosis.

Imaging technology is now available to all patients, allowing the opportunity to personalize care by looking for asymptomatic atherosclerosis. It is known now that inflammation (CANTOS) and the prothrombotic state determine the risk for an atherothrombotic event in the presence of atheroma (22). Individuals with subclinical disease should be considered high risk and their inflammatory and prothrombotic state should be evaluated and managed. These patients should be considered secondary prevention: they already have disease and the goal of therapy should be to prevent progression to mechanical intervention or event. The patients who have already proven they have disease should be classified as tertiary prevention. They are obviously high risk and need optimal medical management to mitigate arterial inflammation and prevent a recidivistic event. The patients who do not demonstrate atherosclerosis after a thorough review of imaging records and no evidence with US of the carotids, femoral, or aorta along with a zero CACS, should be classified as primary prevention. The goal with these patients is to prevent the formation of atheroma. Antithrombotic therapies including aspirin would be unnecessary. Overall their need for prescription medications should be minimal. Thus, this new classification system would mitigate the current harm from medical therapy occurring in the “primary” prevention group. This classification system would move us forward with personalized care which should enhance CVD outcomes (Table 1).

**Table 1:** Proposed binary to ternary classification with treatment goals.

**Patient classification**	**Arterial disease identified**	**History of a CV event or PCI**	**Goal of therapy**
Primary prevention	No	No	Prevent the development of atherosclerosis and vascular inflammation
Secondary prevention	Yes	No	Stabilize plaque and mitigate all sources of arterial inflammation
Tertiary prevention	Yes	Yes	Prevent Recidivism by stabilizing disease and mitigating all sources of arterial inflammation

## Discussion

Traditional cardiology has been a science and study of the lumen of the artery, ensuring that blood flow is maintained. This science has led to miraculous advances in treating end-stage disease and yet, we are still faced with dismal statistics that support the continuation of a rising death and disability rate associated with cardiovascular disease (23). With these compelling statistics as the backdrop, the purpose of this article is to propose two important concepts that will reshape the definition of atherosclerosis and patient CV risk evaluation. A paradigm shift from the definition of vascular disease as a binary approach (those who have had a vascular event and those who have not) to a ternary approach (adding a significant risk category for those who have inflammatory vascular disease but who have not yet had an event). Using this approach, it is appreciated that individuals are vulnerable to vascular events if they are harboring silent and potentially inflammatory atheroma. This approach also demands that we augment many traditional risk assessment tools to identify individuals with vascular disease that lack any lumen diameter changes. Atherosclerosis is much more dynamic than the technology historically designed to find it and evaluate CV risk. An example of this is the common cardiac stress test, which is only considered abnormal if there is a significant amount (>70%) of lumen blood flow obstruction. However, eight-six percent of heart attacks manifest in vessels that are <70% occluded (24). As published previously, it is well-founded that the majority of plaque grows extra-luminally which contributes to the fact that 50% of cardiovascular events are considered to be sudden or unexpected (25). Patients who have normal imaging stress tests frequently have extensive atherosclerosis (26). Additionally, we have known for over three decades that the obstruction causing the lion share of heart attacks and ischemic strokes is a thrombus (27). Using advanced imaging, such as MRI, optical coherence tomography, and near-infrared spectroscopy, it has now been established that the lipid richness of atherosclerosis is the most predictive element of plaque vulnerability and event prediction (28–30). Therefore, appreciating technology that assesses for subclinical lipid-rich plaque is critical for accurate risk assessment for CV events.

The plaque develops in the artery wall creating a vulnerable lesion that can grow silently until the endothelium becomes compromised and allows for a plaque rupture or erosion. A thrombus can form, potentially causing a blockage in blood flow (11). With this understanding, it is imperative that the study of atherosclerosis focus on the wall of the artery where plaque is identified at its most vulnerable state. Most initial coronary events do not occur among those considered “high risk” by risk factor score. Assessing subclinical atherosclerosis at several vascular sites may provide greater insight over traditional risk factors on when assessing for the risk of a CV event (31). Screening methods are recommended and accessible to assess the artery wall for subclinical vascular disease. These include common techniques, such as B-mode ultrasound of the carotid arteries and femoral arteries and coronary calcium scanning (32, 33). This approach to vascular risk assessment using technology to assess for the presence, location, and severity of atherosclerosis is the premise for risk identification. With the focus on the artery wall the earliest most vulnerable lesions can be identified and monitored. Waiting for plaque to rupture and form a subsequent thrombus becomes an emergent situation and has helped drive our current conundrum that CVD remains the leading cause of unexpected death and disability. The microvascular complications of unidentified atherosclerosis lead to diseases of aging, such as erectile dysfunction, chronic heart failure, kidney failure, vascular dementia and peripheral artery disease. These end-stage results of vascular disease are equally debilitating and can lead to decreased quality of life. The current approach waits until the plaque has grown to the point that the lumen is showing change in blood flow, which is ultimately supporting the standard of care approach to treating end-stage disease.

Cardiovascular prevention programs have clinical tests to identify and monitor subclinical plaque in its asymptomatic form (18, 34). Carotid intima-media thickness (cIMT) testing via B-mode ultrasound is an inexpensive method that is safe and simple for evaluating CV risk by measuring the combined thickness of the intimal and medial layers of the artery wall. CIMT testing has the ability to also detect abnormal thickening of the arterial wall, potentially indicating plaque or atheroma's that are associated with accelerated atherosclerotic disease and increased risk for coronary artery disease, myocardial infarction, and stroke (35–37). The cIMT testing of patients to identify and quantify atherosclerotic disease has led to cIMT being adopted as a surrogate endpoint in clinical trials, allowing the efficacy of treatments to be determined much more rapidly than would be possible using CV event or mortality rates alone (29, 38). Perhaps even more compelling from an initial risk assessment and monitoring standpoint, is the lipid-richness of the plaque which can be effectively evaluated over time using MRI imaging, albeit often times more difficult to obtain in the clinical arena. cIMT does have the ability to identify and quantify plaque vulnerability using reproducible measurements of plaque lipid richness into categories of soft, heterogeneous and echogenic (39–43).

Utilizing early disease identification and monitoring has proven successful to identify, stabilize and regress atherosclerosis as validated by reduction of carotid intima media thickness, plaque burden reduction and lipid richness stabilization in the ambulatory clinical setting. Two trials have clinically evaluated this approach to CV Prevention (The BaleDoneen Method). Both trials demonstrate that vascular disease can be identified at its most early and vulnerable state, stabilized, halted, and regressed (18). In both of these clinical trials, patients were assessed for the presence, vulnerability, severity, and location of vascular disease utilizing cIMT testing. Treatments were based on the presence or absence of atherosclerotic plaque and the inflammatory burden of that disease utilizing inflammatory testing to appreciate the vulnerability of the disease. The purpose of Feng's paper was to evaluate the effectiveness of the BaleDoneen Method on traditional CV risk factors (i.e., lipoproteins) and to “illustrate the use of a latent growth-curve analysis in studying trajectories of clinical outcomes and biomarkers in individual patients over time” (18). The analysis evaluated 576 patients at an outpatient ambulatory clinic who received CV care based on this arteriology approach over an 8-years period of time. On average, the mean IMT score decreased by 0.01 mm per year, plaque burden score decreased by 0.17 mm per year (*p* < 0.001 for both). The interpretation was that the BaleDoneen Method is effective in eliciting a positive effect on the atherosclerotic disease process by driving regression of disease in the carotid arteries. The BaleDoneen Method is the study and clinical application of arteriology. The second article, published in partnership with colleagues at Johns Hopkins (34), the BaleDoneen Method demonstrated a decrease in lipid-rich (vulnerable) plaque by 78.4% within the first 2 years of treatment (*p* < 0.0001). After the first 2 years of treatment the lipid-rich plaques continued to decline at reduced rates. These reductions in vulnerable plaque also demonstrated a lack of clinical events in this cohort of 324 patients. The technology embraced by these studies was one of arteriology vs. lumenology, which is a novel concept in traditional cardiovascular disease management.

As previously published by the authors, this approach to prevention is founded in the science of early disease identification The technology is now available to identify asymptomatic arterial disease allowing the shift from treating end-stage disease to one of precision based preventive care (44). In order to appreciate the value of an arteriology approach, the clinician must advance to a vantage point of dividing CV patient risk from a binary approach of primary and secondary prevention to one of a more fluid, yet definable; primary, secondary, tertiary prevention. When this approach is absorbed into clinical practice, the conundrum of vascular treatment decisions, such as aspirin therapy becomes clear. This novel approach to risk assessment is based on the pooling of scientific literature over the past two decades. Appreciating a risk assessment using the arteriology standpoint, patients must first be evaluated for the presence of asymptomatic vascular disease (18, 31, 33, 34, 44).

To articulate the timely importance of utilizing this proposed tiered (primary, secondary, tertiary) approach to risk assessment, we will discuss the recent discussion regarding the risk of utilizing low-dose aspirin therapy in “primary” prevention individuals. We will also articulate the value of our approach to risk identification through the lens of statin therapy for those with vascular disease as a plaque-stabilizing tool. Categorizing patients into three categories, which ultimately helps determine appropriateness of the precision-based medical approach to vascular wellness. Utilizing medical history, patients are determined to be “tertiary” prevention if they have a personal history of a vascular event (heart attack or stroke) or a vascular intervention due to atherosclerosis. “Secondary” prevention is determined when someone is found to have atherosclerosis in the artery wall. This is often determined using non-invasive imaging, such as B-mode ultrasound of the carotid or femoral arteries, ultrasound imaging of the abdominal aorta showing plaque deposits, coronary calcium scanning showing a positive calcium score, an ankle-brachial index (ABI) measurement of <0.95 or any vascular image that shows atherosclerotic plaque. These patients have documented atherosclerosis but have never suffered a vascular event or a need for vascular intervention. Finally, patients who have no history of CV events and are determined to be devoid of atheroma after non-invasive imaging are classified as “Primary Prevention.”

**Figure 1:**
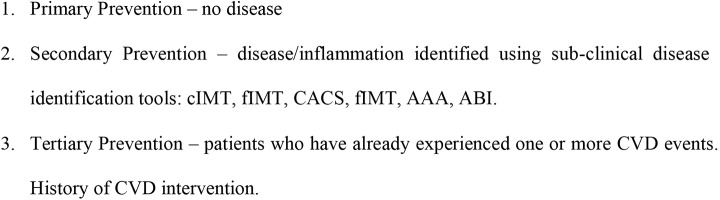
Definition of classification levels.

Additionally, vascular inflammatory testing is utilized to determine the vulnerability of the atherosclerotic disease process in each individual. Setting optimal goals for each tier group is essential to yield optimal health outcomes and demonstrate CV stability in each patient (34) (Figure 1). An approach supported by identifying vascular disease allows for clear need for plaque stabilizing therapies, such as statin therapy. The concept is gaining traction as demonstrated in Ramos' research showing that statins in low-risk patients with an ABI <0.95 prevented vascular events (45). Statin therapy is indicated in patients determined to have a 10-years Framingham Risk Score >7.5 (46). However, statin therapy is also indicated to reduce vascular inflammation as documented in the Jupiter Trial showing the benefit of statin therapy in patients with low-density lipoprotein (LDL) LDL <130 mg/dL (47). In 2013, it was demonstrated that the benefit of statin therapy was best noted by the ability to lower Lp-PLA2 rather than simply lowering LDL (48).

Additionally, Puri et al. demonstrated that statins can increase coronary calcification (49). Using the BaleDoneen Method approach to risk assessment, this supports the concept of stabilizing atherosclerosis and removing the vulnerable elements of plaque (34, 50). Appreciating the body of evidence that has been previously demonstrated, it is imperative to refrain from a binary approach to risk assessment and categorize each patient using a ternary system approach to assessment of disease and categorize each as primary (no plaque), secondary (plaque/inflammation) and tertiary (symptomatic lesions). This approach takes established treatments, such as statins, to the forefront for individuals with vascular disease rather than simply guessing of their disease burden using risk factors alone.

The same approach can be taken with low-dose aspirin (ASA) therapy, eliminating the clinical confusion of determining when to use low-dose aspirin in patients who have not had a vascular event. Using our ternary definition, all patients deemed to have atherosclerosis (secondary and tertiary) should be placed on low-dose aspirin unless contraindicated for bleeding risk.

“The effectiveness of low-dose ASA for people who have already suffered one or more heart attacks or strokes remains undisputed. Daily aspirin is prescribed for all people who fall into the traditional definition of ‘secondary prevention.' However, using this historic model, the ‘primary prevention' individual may have asymptomatic vascular disease and would also benefit from low-dose aspirin therapy. A plethora of studies have demonstrated that ASA reduces rates of recidivistic events thus allowing the benefit to outweigh the risk, particularly bleeding associated with aspirin therapy” (51).

Current guidelines are based on the Framingham Risk Score (FRS) which provides recommendations for aspirin's use in primary prevention which is based on a risk-factor analysis. The U.S. Preventive Services Task Force (USPSTF) also discussed aspirin's utility for colon cancer prevention. The USPSTF only recommends the drug for people who are ages 50–69, have a 10% or higher 10-years risk for CVD and are at no increased risk for bleeding. The USPSTF considers the evidence insufficient to recommend low-dose ASA for people under age 50 or over age 69, regardless of the magnitude of their risk (51).

The role of ASA in primary prevention has remained controversial. Five RCTs conducted between 1988 and 2003 linked aspirin use to a 32% reduction in first-time heart attacks. Since then additional RCTs have been published with inconsistent findings, leading to inconsistent guidelines, with medical societies and government agencies in the U.S. and Europe recommending both for and against low-dose aspirin for primary prevention. Here are key findings from the three latest RCTs:

To highlight the relevant value of our ternary definition of CVD risk, let's examine three recent trials with aspirin use in people who have not had vascular events. The ASPREE, ASCEND, and ARRIVE trials all demonstrate a concern with prescribing low dose aspirin for CVD risk prevention in “primary prevention” patients (no previous recorded heart attack). Although rates of CVD events were lower in the aspirin group in all three trials, the bleeding risk challenged the statistical significance of the findings. This current recommendation for aspirin use is reserved for those with known cardiovascular disease or risk profiles that demonstrate a moderate to high-risk FRS score (52–55).

Given the conflicting findings of 30 years of research on the role of low-dose ASA in primary prevention, and recent RCTs raising questions as to whether the benefits outweigh the harms, what should patients and medical providers conclude? Using our proposed BaleDoneen Method Ternary approach to CVD risk determination, the decision about low-dose aspirin use is actually about proper patient selection. While the standard of care divides patients into two groups based on whether or not they have experienced a CV event, we recommend a precision-medicine, three-tiered approach that starts with a comprehensive evaluation that includes laboratory testing and vascular imaging. We also recommend that patients who are being considered for low-dose ASA for prevention of CVD or CV events be screened for aspirin resistance. In a meta-analysis of 1,813 patients with CVD from twelve prospective studies, the average prevalence of aspirin resistance was 27%. Aspirin-resistant patients were also found to have nearly quadruple the rate of CV events, compared to aspirin-responsive patients. Another recent study revealed that the odds ratio for recurrent stroke is 14 times higher in non-responders to ASA. These findings highlight the paramount importance of determining the patient's ASA status before initiating a therapy that may fail to protect a large proportion of patients (51).

We consider the current controversy about low-dose ASA for “primary prevention” a valuable opportunity for patients and providers to shift away from using traditional “risk-factor profiling” to guide treatment decisions to being guided by the disease/inflammation approach employed by the BaleDoneen Method. Two recent peer-reviewed studies have demonstrated our precision-medicine approach to prevention and treatment can effectively detect, stabilize and reverse CVD, helping patients avoid heart attacks and strokes even if they have previously suffered one or more of these events (18, 34).

## Conclusion

Migrating from a binary approach to risk assessment to a ternary model of disease identification allows for individualized, optimal management. Using a disease/inflammatory approach has been proven to identify, stabilize, and regress atherosclerosis while adding understanding to the progression of vascular disease (34, 56). Our previously published results show the beneficial effect of comprehensive, evidence-based management on subclinical atherosclerosis and vulnerable plaque. This approach does not mitigate the value of utilizing standard risk factor identification but rather augments it for the benefit of the individual patient.

In order to suffer a CV event, the presence of atherosclerosis is necessary. Waiting for the atherosclerotic disease process to rupture and cause an event is continuing to drive the morbidity and mortality data associated with CV disease. We have affordable safe accessible imaging modalities to evaluate each person for the presence of atheroma. This action should be at the center of any prevention program. Using a binary system, demands that we wait until someone has had a CV event or a CV intervention; thus, labeling them secondary prevention. In the new system they would be referred to as tertiary prevention; most certainly deserving of comprehensive management of all CV risk factors to mitigate recidivism risk. Patients who are discovered to have arterial disease albeit without any history of intervention or events would be classified as secondary prevention. They possess the pre-requisite for needing an intervention or suffering a CV event. They need comprehensive management of all known CV risk factors to extinguish any arterial inflammation and to minimize a pro-thrombotic state. In order to stabilize plaque in both the secondary and tertiary prevention categories, patients will assuredly require pharmaceutical intervention which needs to be handled with care and balance the potential for possible harm. After a comprehensive arterial health evaluation, the patients who fail to demonstrate any atheroma would be classified as primary prevention with a goal of management to stop them from ever developing atherosclerosis. Just like the secondary and tertiary patients, the primary prevention patients will require regular monitoring of their arterial inflammatory state and monitored over time. Non-pharmaceutical therapies would be the preferred management as they generally carry less risk. Theoretically, assuming the search for subclinical disease was sufficient, these individuals do not have any atheroma's which generates very low risk for a CV intervention or event. This ternary approach supports a precision-based risk assessment that allows for the optimal care of in the unique, individual patient with the aim to live a life free of CV events.

## Author Contributions

All authors listed have made a substantial, direct and intellectual contribution to the work, and approved it for publication.

## Conflict of Interest

The authors declare that the research was conducted in the absence of any commercial or financial relationships that could be construed as a potential conflict of interest.
